# Cervical compressive myelopathy in Africa: a systematic review of clinical presentation, diagnostic pathways, surgical management, and outcomes

**DOI:** 10.1186/s12883-026-05106-x

**Published:** 2026-06-29

**Authors:** Godswill Uzoechina, Abdulbasit Opeyemi Muili, Prabuddha Bajracharya, Williams Ajayi, Vidushi Joshi, Teslim Ibrahim, Kasali AbdulGafar Adesola, Abdulsalam Abdulsalam

**Affiliations:** 1 Faculty of Clinical Sciences, University of Nigeria Teaching Hospital, Enugu, Nigeria; 2 Department of Medicine, Ladoke Akintola University of Technology, Ogbomosho, Nigeria; 3 Patan Academy of Health Sciences, Lalitpur, Nepal; 4 Department of Medical Laboratory Science, College of Health Sciences, University of Ilorin, Ilorin, Nigeria; 5 China Medical University, Shenyang, China; 6 Faculty of Clinical Sciences, University of Ilorin, University of Ilorin Teaching Hospital, Ilorin, Nigeria

**Keywords:** Cervical myelopathy, Degenerative cervical myelopathy, Sub-Saharan Africa, Spinal decompression surgery, Diagnostic imaging (MRI)

## Abstract

**Background:**

Degenerative cervical myelopathy (DCM) is the leading cause of non-traumatic cervical spinal cord dysfunction globally; however, broader cervical compressive myelopathies remain poorly characterised across African settings. This systematic review evaluated the clinical presentation, diagnostic pathways, surgical management, and outcomes of cervical myelopathy across African settings.

**Methods:**

This review was conducted according to PRISMA 2020 guidelines. PubMed, African Journals Online (AJOL), Google Scholar, and African neurosurgical registries were searched from inception to 25 December 2025. Both degenerative and non-degenerative compressive cervical myelopathies were eligible where cervical cord compression was clearly described. Data were synthesised narratively because of substantial methodological and clinical heterogeneity. Risk of bias was assessed using Joanna Briggs Institute (JBI) appraisal tools.

**Results:**

Fifteen studies involving 315 patients from four African sub-regions met inclusion criteria. Degenerative cervical spondylotic myelopathy constituted the predominant aetiology across major surgical cohorts, although several studies included mixed myelopathy populations or rarer compressive causes such as fluorosis-associated OPLL and CPPD-related ligamentum flavum calcification. Patients consistently presented late, with prolonged symptom duration (12–48 months) and advanced neurological impairment, commonly Nurick grade ≥ 3. Motor deficits and gait disturbance were the dominant presenting features, while sphincter dysfunction reflected advanced disease. Diagnostic pathways evolved from plain radiography and myelography to increasing MRI utilisation; however, MRI scarcity remained a major barrier contributing to delayed diagnosis. Surgical decompression, via anterior cervical discectomy and fusion or posterior laminectomy, produced neurological improvement in 50–97.6% of patients. Earlier presentation and lower preoperative severity were the strongest predictors of favourable recovery. No included study demonstrated low overall risk of bias.

**Conclusion:**

Cervical myelopathy in Africa is characterised by delayed presentation and limited diagnostic resources, yet surgical decompression appears feasible and is associated with neurological improvement in reported cohorts. Expansion of MRI access, earlier referral systems, and prospective multicentre registries are required to improve outcomes and strengthen the evidence base.

**Supplementary Information:**

The online version contains supplementary material available at https://doi.org/10.1186/s12883-026-05106-x.

## Introduction

### Background

Cervical spondylotic myelopathy (CSM), or degenerative cervical myelopathy (DCM), is the leading cause of non-traumatic spinal cord dysfunction in adults and a major source of progressive neurological disability worldwide [1–3]. It arises from age-related degenerative changes of the cervical spine: disc degeneration, osteophyte formation, ligamentum flavum hypertrophy, and congenital canal narrowing, resulting in progressive spinal cord compression and chronic ischemic injury [4].

Although radiological evidence of cervical cord compression is common, clinical disease is substantially less frequent. MRI-based studies estimate that up to 24.2% of adults may demonstrate asymptomatic cervical cord compression, while only approximately 2–3% develop symptomatic myelopathy [1, 5]. This disparity reflects the often prolonged subclinical phase of disease progression, during which early detection could potentially prevent irreversible neurological injury.

Clinically, CSM presents insidiously with gait disturbance, hand dysfunction, sensory deficits, and upper motor neuron signs such as hyperreflexia and spasticity, with advanced disease leading to sphincter dysfunction [6]. However, symptom recognition is frequently delayed or missed in primary care settings due to its non-specific presentation. As a result, diagnostic delay exceeding 2 years from symptom onset has been reported in observational cohorts [7, 8]. Such delays are clinically relevant, as longer symptom duration is consistently associated with poorer postoperative neurological recovery and reduced functional improvement following decompression surgery [8, 9].

In low- and middle-income countries (LMICs), particularly in sub-Saharan Africa, the burden of CSM is intensified by health system limitations. Patients often present late with advanced neurological deficits due to weak referral systems and limited access to neurosurgical care [10]. Diagnostic capacity is also constrained: while MRI is the gold standard for confirming cord compression and intramedullary signal change, its availability remains limited, leading to reliance on plain radiography and computed tomography, which detect disease at more advanced stages [11, 12].

Regional data indicate that compressive myelopathies, including CSM, spinal tuberculosis, and metastatic disease, account for over 48% of non-traumatic myelopathy cases in sub-Saharan Africa [10]. Outcomes remain poor, with persistent disability reported in approximately 50% of patients and in-hospital mortality approaching 10% in some series [13, 14]. In Ethiopia, clinically significant CSM has been reported in approximately 5.6% of patients with cervical spondylosis, underscoring its relevance in African clinical populations [15].

Despite this burden, CSM remains under-characterised in African settings, with most evidence derived from small, retrospective, single-centre studies, in contrast to the large prospective registries available in high-income countries.

Although degenerative cervical spondylotic myelopathy represents the predominant form of cervical cord compression globally, other compressive aetiologies, including ossification disorders, metabolic disease, and ligamentous calcification, may also contribute to cervical myelopathy in African clinical practice. Because African neurosurgical literature frequently reports mixed compressive cohorts, this review adopts a broader cervical compressive myelopathy framework while recognising degenerative CSM/DCM as the dominant subtype.

### Knowledge gap

Although cervical myelopathy represents a significant cause of disability in African populations, the current evidence base remains fragmented, largely derived from small, retrospective, single-centre studies [10, 16]. There is no consolidated synthesis that specifically evaluates the clinical, diagnostic, and therapeutic characteristics of CSM within the African or broader LMIC context.

Three major knowledge gaps are evident:

(i) Clinical severity at presentation
  A consistent theme across African neurosurgical literature is delayed presentation, with patients frequently arriving at tertiary centres with advanced neurological deficits, including severe gait impairment, long-tract signs, and sphincter dysfunction. These delays are driven by both patient-level factors (low awareness, delayed healthcare-seeking) and system-level barriers (limited access to specialists, referral inefficiencies). However, the true distribution of disease severity at presentation across settings remains poorly quantified and inconsistently reported.
(ii) Imaging evolution and diagnostic pathways
  MRI remains the definitive imaging modality for diagnosing CSM due to its ability to directly visualise cord compression and intramedullary signal changes. However, in many African healthcare systems, access to MRI is limited or absent, particularly outside tertiary centres. Consequently, clinicians rely on stepwise imaging pathways involving plain radiography and computed tomography, which may detect only late structural changes. There is currently no systematic synthesis describing how imaging modalities are utilised across African settings, nor how delayed or absent MRI access influences stage at diagnosis, surgical timing, and neurological outcomes.
(iii) Surgical outcomes in resource-limited environments
  Surgical decompression is the mainstay of treatment for progressive CSM, with well-established benefits in high-income countries. However, in resource-limited African settings, outcomes are influenced by delayed presentation, limited neurosurgical workforce density, inconsistent access to spinal instrumentation, and variable postoperative rehabilitation services. Despite this, published outcome data remain sparse, heterogeneous, and largely descriptive, with no comprehensive synthesis evaluating neurological recovery, complication rates, or long-term functional outcomes in these environments.

### Objective

This systematic review aims to comprehensively evaluate the existing literature on cervical myelopathy in African settings in order to address these critical gaps in knowledge. Specifically, the review will:

- Characterise the clinical spectrum of cervical myelopathy, including severity and patterns of neurological deficit at presentation in LMIC settings.
- Evaluate diagnostic imaging pathways (X-ray, CT, and MRI), including accessibility, utilisation patterns, and their impact on diagnostic timing and disease stage.
- Assess surgical management strategies and postoperative neurological and functional outcomes in resource-limited environments.
- Critically appraise the methodological quality of available evidence to inform future research priorities and clinical guideline development.

## Materials and methodology

### Protocol and reporting

This systematic review was conducted in accordance with the Preferred Reporting Items for Systematic Reviews and Meta-Analyses (PRISMA 2020) guidelines [17]. Due to expected heterogeneity in study design, patient populations, imaging modalities, and outcome reporting, a meta-analysis was not feasible. Instead, findings were synthesised using a structured narrative synthesis approach in line with established methodological guidance [18]. The review protocol was not prospectively registered in PROSPERO, which represents a limitation.

### Eligibility criteria

Studies were included if they met the following criteria:

- Population and condition
  - Adults and/or mixed-age populations with cervical spondylotic myelopathy (CSM) or degenerative cervical myelopathy (DCM)
  - Studies where cervical myelopathy data could be clearly extracted
- Study designs
  - Retrospective observational studies
  - Prospective cohort studies
  - Case series
  - Case reports (included only for rare or atypical etiologies)
- Outcomes
  - Studies reporting at least one of the following:
    - Clinical presentation or neurological status
    - Imaging findings (X-ray, CT, or MRI)
    - Surgical intervention and outcomes
    - Functional or neurological outcome scores (e.g., mJOA, Nurick, Odom)
- Exclusion criteria
  - Studies exclusively reporting thoracic or lumbar myelopathy without cervical data
  - Review articles, editorials, commentaries, and conference abstracts without full data
  - Animal or experimental studies

### Search strategy

A comprehensive literature search was conducted to identify studies reporting cervical spondylotic myelopathy (CSM) or degenerative cervical myelopathy (DCM) in African and other low-resource settings. The search covered from inception to 25 December 2025, reflecting contemporary diagnostic and surgical practice, particularly the increasing availability of CT and MRI imaging. All studies included in the final synthesis were published or electronically available before the final search date.

Electronic searches were performed in PubMed, African Journal Online (AJOL), and Google Scholar, supplemented by targeted searches of African trauma and neurosurgical registries. To maximise sensitivity, the search strategy combined MeSH terms (where available), database-specific subject headings, and free-text keywords, tailored to each database interface. The strategy was developed iteratively and was peer-reviewed by an information specialist in accordance with PRESS (Peer Review of Electronic Search Strategies) guidelines.

The core search concepts included:

- Condition terms:
  “cervical spondylotic myelopathy” OR “degenerative cervical myelopathy” OR “cervical myelopathy”
- Anatomical/clinical context:
  “spinal cord compression” OR “cervical spine degeneration”
- Setting and geography:
  “Africa” OR names of individual African countries OR “sub-Saharan Africa”
- Healthcare system and management context:
  “neurosurgery” OR “spinal surgery” OR “surgical decompression”
- Outcomes and clinical severity:
  “outcome” OR “neurological recovery” OR “Nurick” OR “mJOA” OR “disability”

Boolean operators (AND/OR) were applied appropriately to combine concepts and refine search sensitivity. Studies published in English or French were eligible for inclusion. French-language studies were screened and extracted using translated full texts where required. Complete database-specific search strategies are provided in Supplementary File 1.

The database and supplementary searches identified 4,748 records, including 4,660 from Google Scholar and 88 from PubMed. After removal of 1,088 duplicate records, 3,660 titles and abstracts underwent screening, of which 3,594 were excluded. Sixty-six reports were sought for retrieval, and 60 full-text articles were assessed for eligibility. Forty-five reports were excluded following full-text review, including non-original publications (*n* = 24), studies not specifically addressing cervical spondylotic or compressive myelopathy (*n* = 15), and studies without extractable clinical or outcome data (*n* = 6). Fifteen studies met the final inclusion criteria and were included in the qualitative synthesis.

No additional eligible studies were identified through AJOL, registry searches, or manual reference screening beyond those captured in the primary database searches.

In addition to database searching, hand-searching of reference lists from all eligible studies was performed to identify additional relevant publications. Although databases such as Embase and Scopus are commonly included in systematic reviews, the search strategy prioritised databases and repositories most likely to index African clinical and neurosurgical literature, where indexing is often inconsistent across large bibliographic platforms.

No additional studies meeting the inclusion criteria were identified through supplementary screening.

### Study selection

**Fig. 1:**
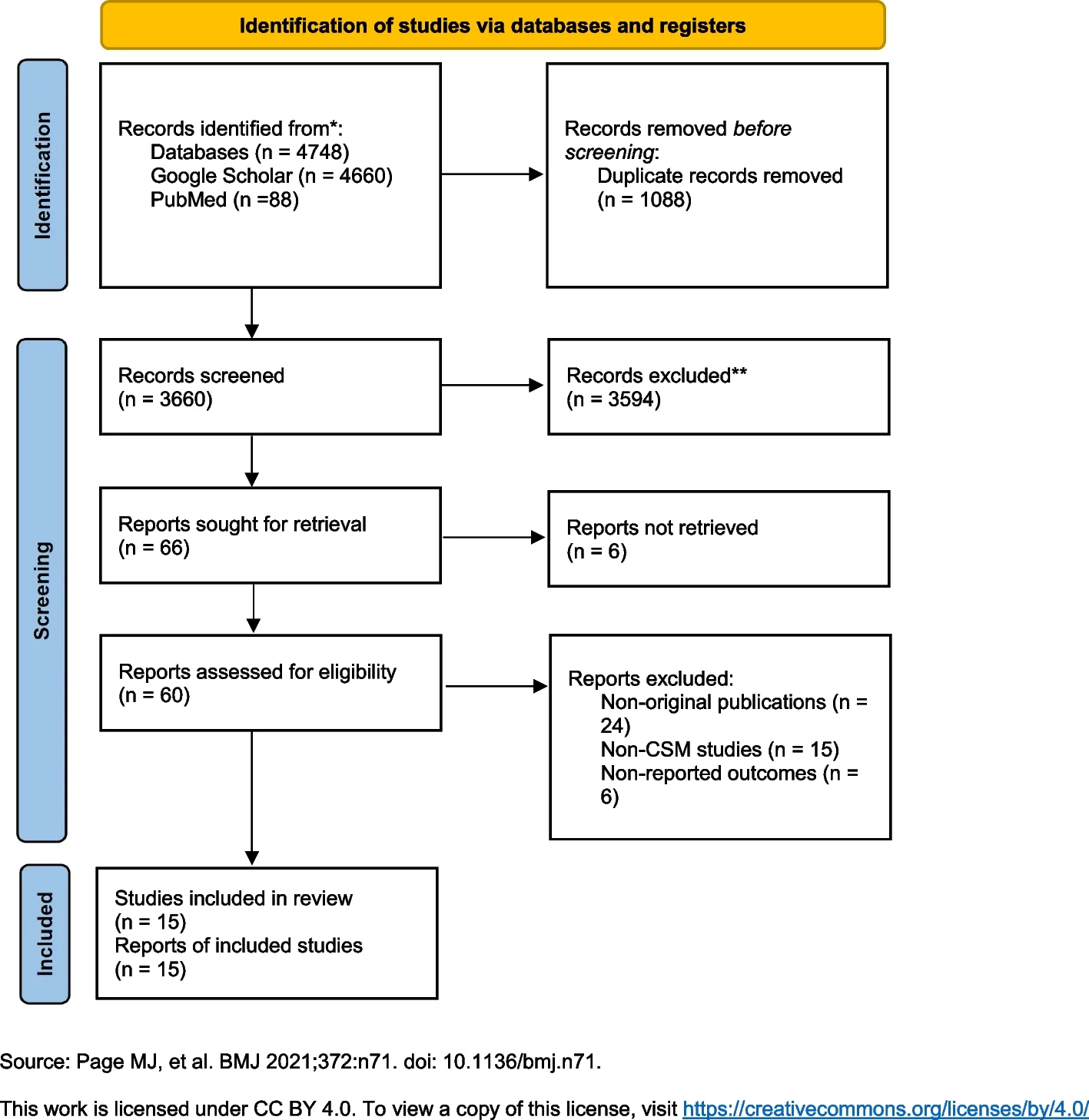
PRISMA flow diagram showing the process of study identification, screening, eligibility assessment, and final inclusion in the systematic review

Title and abstract screening were performed independently by two reviewers using predefined eligibility criteria. Full-text articles considered potentially eligible underwent independent review. Disagreements were resolved through discussion and consensus. The study selection process is summarised in Fig. 1.

### Risk of bias assessment

Risk of bias was assessed using the Joanna Briggs Institute (JBI) critical appraisal tools, selected according to study design [19]:

- Case series: JBI Case Series Checklist
- Cross-sectional studies: JBI Analytical Cross-Sectional Checklist
- Case reports: JBI Case Report Checklist

Studies were then classified into three interpretive levels for synthesis:

- Low risk of bias: Methodologically stronger observational cohorts with structured outcome assessment and relatively complete follow-up
- Moderate risk of bias: Retrospective or prospective series with incomplete follow-up, limited imaging standardisation, or absence of comparators
- High risk of bias: Single case reports, very small series, or studies with substantial missing or non-specific outcome data

This classification was used for the interpretation and weighting of evidence, not for exclusion.

### Data extraction

Data were extracted independently using a standardised extraction framework. The following variables were collected:

- Study characteristics
  - Author, year, country, and study design
  - Sample size (total and cervical myelopathy subgroup)
- Patient characteristics
  - Age and sex distribution
  - Symptom duration prior to presentation
- Clinical features
  - Neurological presentation (motor, sensory, gait disturbance, sphincter involvement)
- Aetiology
  - Degenerative CSM/DCM
  - Other compressive causes (e.g., ossification, infection, metabolic disease)
- Imaging modality
  - Plain radiography
  - Computed tomography (CT)
  - Magnetic resonance imaging (MRI)
  - Access limitations or substitution of modalities
- Management
  - Surgical approach (anterior, posterior, combined, decompression ± fusion)
  - Non-surgical management where applicable
- Outcomes
  - Neurological scores (mJOA, Nurick, Odom)
  - Clinical improvement (qualitative or quantitative)
  - Complications and mortality
  - Duration of follow-up
- Study limitations
  - As reported by the authors and independently assessed

### Data synthesis

Given substantial heterogeneity in study design, imaging availability, surgical techniques, and outcome measures, data were synthesised using a thematic narrative synthesis approach.

Findings were structured into five analytical domains:

1. Clinical presentation and severity at diagnosis
2. Aetiological patterns of cervical myelopathy
3. Imaging pathways and diagnostic evolution (X-ray → CT → MRI access patterns)
4. Surgical management strategies in resource-limited settings
5. Neurological and functional outcomes following treatment

Case reports and very small series were analysed qualitatively only and were not integrated into aggregated outcome interpretations.

The study selection process and reasons for exclusion are detailed in Fig. 1 (PRISMA flow diagram).

## Results

### Study selection

In this systematic review, we have identified 15 studies that meet the predefined inclusion criteria. In total, they represent 315 patients with cervical myelopathy across the African continent. These studies span four African sub-regions: West Africa (Nigeria [14, 20–22], and Ghana [23]), Central Africa (Cameroon [24, 25], Gabon [26], and Togo [27]), East Africa (Ethiopia [28], and Malawi [29]), and North Africa (Egypt [30, 31], and Tunisia [32, 33]). No eligible studies from Southern Africa were identified. Table 1 summarises the characteristics of included studies:

**Table 1:** Characteristics of included studies on cervical myelopathy in Africa

First Author, Year	Country	Study Design	Sample Size (n)	Key Clinical Features	Aetiology	Surgical Intervention	Main Outcomes	Risk of Bias
Loembe et al., 2004 [26]	Gabon	Retrospective case series	18	Spastic tetraparesis, gait disturbance	Degenerative CSM	Anterior decompression & fusion	55.6% complete recovery, 38.9% partial (Nurick)	High
Figuim et al., 2024 [25]	Cameroon	Multicentre retrospective	52	Gait problems (100%), motor deficit (90%)	Degenerative CSM (67% multilevel)	Posterior (86.5%), anterior (13.5%)	82% improved at 1 yr	High
Ojo & Ikwuegbuenyi, 2020 [14]	Nigeria	Prospective cohort	43	Tetraparesis, unsteady gait, falls	Degenerative CSM	ACDF (51%), laminectomy (33%)	97.6% improved; mean Nurick 3.2 → 1.14	Moderate
Nyada et al., 2020 [24]	Cameroon	Cross-sectional	39	Pyramidal syndrome (100%), tetraparesis (72%)	Degenerative CSM	Posterior laminectomy (79.5%)	Acceptable perioperative outcomes	High
Andrews et al., 2003 [23]	Ghana	Retrospective series	40	Motor deficits, hyperreflexia	Degenerative CSM	Non-instrumented ACDF	86% excellent/good (Odom’s); Nurick 2.3 → 1.3	High
Elbhrawy et al., 2021 [30]	Egypt	Prospective cohort	25	Myelopathy (48% severe)	Degenerative CSM	ACDF (52%) or posterior fusion (48%) + IONM	Significant mJOA improvement at 3 & 12 mo	Moderate
Kassegne et al., 2013 [27]	Togo	Retrospective	9	Walking disorder (97%), sphincter dysfunction (82%)	Degenerative CSM	Laminectomy	56% improved (hospital discharge)	High
Olarinoye-Akorede et al., 2015 [20]	Nigeria	Retrospective radiological	32	Neck pain, gait abnormality, paresis	Degenerative CSM	Surgery for moderate–severe cases	42.1% of cervical MRIs; C3/4 most affected	High
Fidele & Amanuel, 2016 [28]	Ethiopia	Retrospective	16*	Weakness (100%), sphincter issues (57%)	Degenerative (15.2% of myelopathies)	Laminectomy (62.5%)	50% surgical improvement	High
Mohammed et al., 2025 [31]	Egypt	Case report	2	Gait disturbance, hand clumsiness	CPPD (calcified LF)	Posterior laminectomy ± fusion	Both improved to Nurick 1	High
Younes et al., 2008 [33]	Tunisia	Case report	1	Spastic tetraparesis, dysaesthesia	Fluorosis + OPLL	Multilevel laminectomy	Pain & sensory improvement; motor residual	High
Guesmi et al., 2005 [32]	Tunisia	Case report	1	Spastic tetraparesis	Calcified ligamentum flavum	C3–C4 laminectomy	Complete resolution	High
Ogunniyi et al., 1995 [21]	Nigeria	Retrospective	31**	Cervical myelopathy	Likely degenerative	NR	Limited data	High
Adeloye, 1999 [29]	Malawi	Case series	5	Cervical myelopathy	Presumed degenerative	Laminectomy	Limited data	Very High
Rabiu et al., 2023 [22]	Nigeria	Retrospective ICU review	1***	Severe myelopathy	Severe CSM	NR	ICU mortality case (1.2%)	High

^*^Cervical degenerative subgroup extracted from a larger mixed myelopathy cohort (*n* = 105)

^**^Whole cohort ratio

^***^Single CSM case within a broader ICU mortality series

Of the 315 included patients, the majority were derived from degenerative cervical spondylotic myelopathy cohorts, while smaller numbers originated from mixed myelopathy populations, isolated case reports, or atypical compressive aetiologies.

### Study design and methodological quality (JBI assessment)

**Fig. 2:**
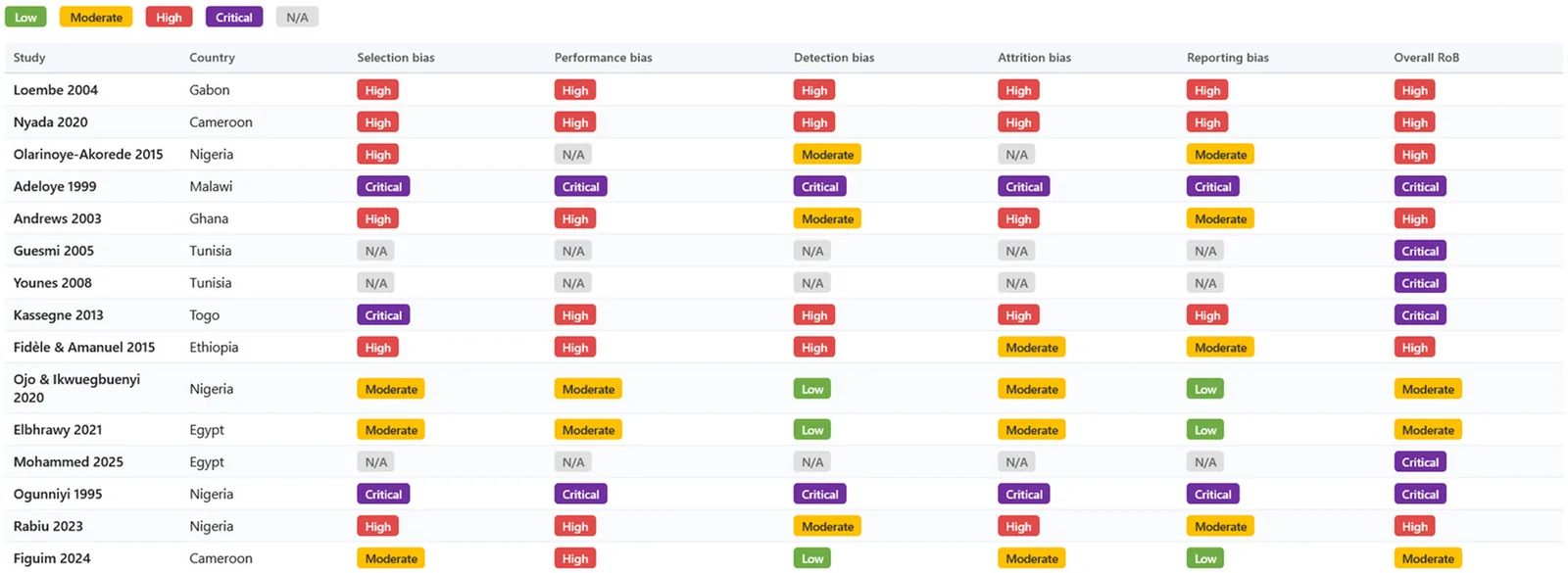
Summary of methodological quality assessment using JBI critical appraisal domains

Study designs were heterogeneous and predominantly retrospective, accounting for more than 70% of included studies all employed retrospective or cross-sectional descriptive designs, conferring a high risk of bias across this majority [20–27]. Two studies employed a prospective cohort design [14, 30], and were accordingly judged to carry a moderate risk of bias by virtue of predefined outcome assessments at standardised postoperative intervals. Three studies were single-case or two-case reports [31–33], and two additional studies provide only qualitative or incidental cervical myelopathy data [22, 29], all classified as carrying a very high risk of bias. No study was judged to be at low overall risk of bias. The RoB is summarised in Fig. 2

### Clinical presentation

#### Core neurological pattern

Motor deficits—manifesting as tetraparesis, paraparesis, or in extreme cases tetraplegia—constituted the single most consistently reported clinical finding, present in approximately 70–100% of patients across surgical cohorts where this was extractable. Spastic tetraparesis was the dominant motor syndrome, documented in all 18 patients by Loembe et al. [26], in 71.8% by Nyada et al. [24], and in both cases reported by Mohammed et al. [31]. Ojo & Ikwuegbuenyi [14] described weakness of all limbs with muscle wasting as cardinal features in their 43-patient Nigerian cohort.

Gait disturbance was universal across the surgical series. Figuim et al. [25] recorded gait problems in 100% of their 52-patient Cameroonian multicentre cohort; Ojo & Ikwuegbuenyi [14] reported unsteady gait and frequent falls as near-universal presenting features; and Kassegne et al. [27] documented walking disorders in 97% of their nine cervical cases in Togo.

Sensory deficits—encompassing paraesthesiae, numbness, dysaesthesia, and proprioceptive loss—were variably reported, with frequencies ranging from approximately 40% to 80% across studies providing this data. Kassegne et al. [27] documented sensory disorders in 47%, Fidele & Amanuel [28] in 45.7% of their degenerative cervical subgroup, and Younes et al. [33] described dysaesthesia in all four limbs in their fluorosis case. Olarinoye-Akorede et al. [20] described paraesthesia, tingling, and loss of proprioception among the cardinal presenting features in their Nigerian radiological series.

Sphincter dysfunction was consistently described as a late or advanced feature, reflecting delayed health-seeking behaviour. Reported frequencies ranged from approximately 11% to 82%: Figuim et al. [25] noted sphincter disorders in 11.38% of their cohort, while Kassegne et al. [27] documented this in 82% and Fidele & Amanuel [28] in 57.1% of the degenerative cervical subgroup. This wide range likely reflects both differences in disease stage at presentation and variation in the rigour of sphincter assessment across studies. Additional presenting symptoms across cohorts included cervicalgia [25, 26], cervicobrachialgia and cervicobrachial neuralgia [24, 25], and radicular pain [27].

#### Disease severity and stage at presentation

**Fig. 3:**
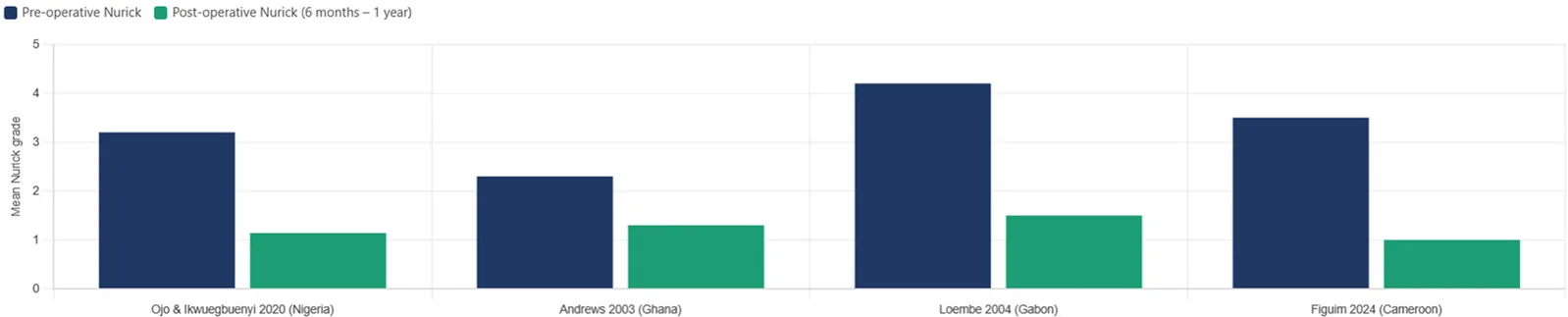
Nurick grade: pre- vs post-surgical (quantitative data; 4 studies). Pre- and postoperative Nurick grades across included studies reporting extractable data (*n* = 4 studies; total patients = 153). Values represent study-reported group means or distributions. No pooled estimate or error bars were calculated due to methodological and clinical heterogeneity between studies

The majority of patients in all cohorts presenting reported data presented with advanced myelopathy equivalent to Nurick grade ≥ 3. In the Gabon series [26], preoperative Nurick grades were III (*n* = 9), IV (*n* = 6), and V (*n* = 3). Figuim et al. [25] reported Nurick grade 3 in 38.46% and grade 4 in 32.69% of their 52-patient series, designating Nurick grade ≥ 2 as the surgical threshold. Ojo & Ikwuegbuenyi [14] documented a mean preoperative Nurick grade of 3.2 in their Nigerian cohort, with 91% classified as late presenters (> 3 months from symptom onset). Elbhrawy et al. [30] found 48% of their Egyptian cohort classified as severe by modified JOA (mJOA) criteria, with a mean preoperative mJOA of 11.84. Figure 3 summarises Nurick grade: pre- vs post-surgical for the four studies.

**Fig. 4:**
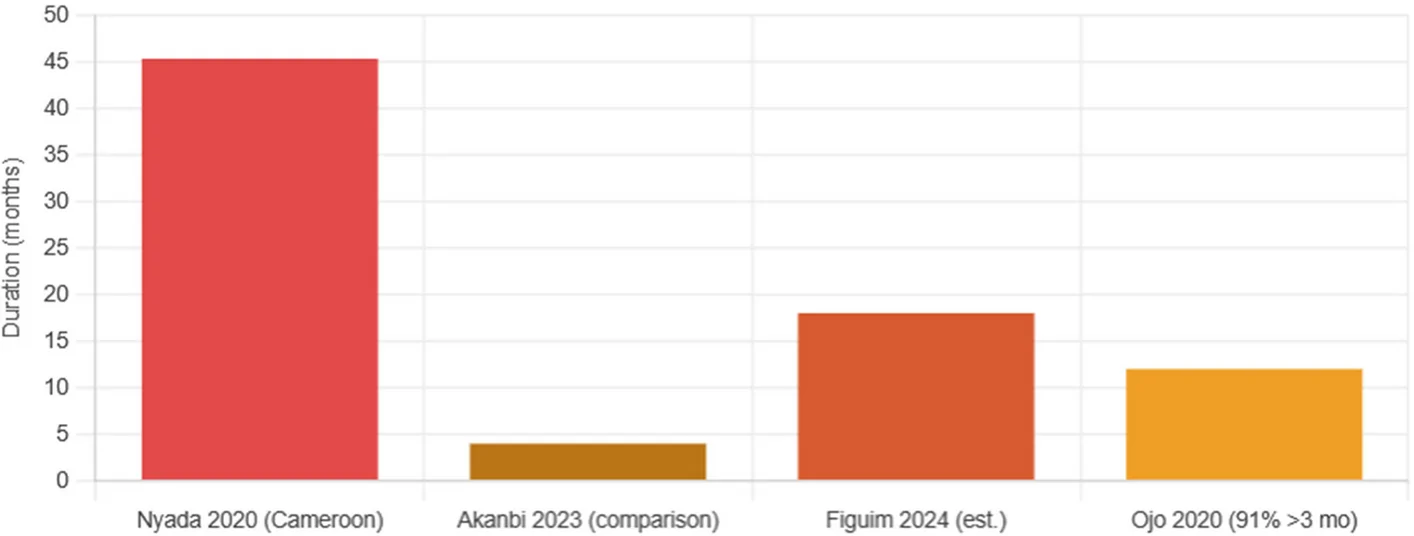
Symptom duration at presentation (months). Symptom duration at presentation (months) across included studies reporting extractable data. Values represent study-level mean symptom duration or reported ranges. Total patients contributing to extractable data: *n* ≈ 118. No error bars or pooled estimates were generated due to high inter-study heterogeneity and inconsistent reporting formats

Mean symptom duration before presentation was prolonged in all studies reporting this variable. Nyada et al. [24] documented a mean symptom duration of 45.3 months in their Cameroonian cohort. Younes et al. [33] described a 3-year progressive history in their fluorosis case. Ojo & Ikwuegbuenyi [14] reported that 91% of patients were late presenters. Across all contributing studies, mean symptom duration before surgical consultation ranged from approximately 12 to 48 months. Figure 4 shows the symptom duration at presentation (months).

### Aetiology and diagnostic distribution

Degenerative cervical spondylotic myelopathy (CSM) was the leading aetiology across all surgical series. Every major cohort confirmed CSM as the dominant diagnosis. This pattern appeared in Gabon [26], Cameroon [24, 25], Nigeria [14, 20, 21], Ghana [23], Egypt [30], and Togo [27].

Multilevel cervical disease was frequent across studies. Figuim et al. [25] reported that 67.3 per cent of patients had involvement of three or more levels. Andrews et al. [23] found 90 per cent of surgical fusions at C4-5 and C5-6. Olarinoye-Akorede et al. [20] identified C3/4 as the most affected level at 38.1 per cent, followed by C4/5 at 30.9 per cent. These findings show a consistent pattern of multi-level degenerative compression rather than isolated single-level disease.

Mixed myelopathy cohorts also reflected a significant contribution from degenerative disease. In Ethiopia, degenerative cervical and spinal myelopathy accounted for 15.2 per cent of cases [28]. In Togo, it represented 23 per cent of slow cord compression cases and ranked second after malignant tumours [27].

Less common aetiologies appeared across isolated reports but carried clinical importance. CPPD-related calcification of the ligamentum flavum caused cervical cord compression in Tunisia [32] and in two Egyptian cases where it emerged as the main cause of symptomatic calcified ligamentum flavum [31]. Skeletal fluorosis with ossification of the posterior longitudinal ligament was reported in Tunisia by Younes et al. [33], involving multilevel disease from C2 to C5 with associated ligamentum flavum ossification. Rabiu et al. [22]also identified CSM as the primary diagnosis in 1.2 per cent of neurosurgical ICU deaths in Nigeria, highlighting its severity in advanced cases.

### Diagnostic imaging trends

A clear temporal evolution in diagnostic imaging modalities was evident across the included studies, broadly corresponding to their chronological span (1995–2025) and the uneven diffusion of advanced neuroimaging across African health systems.

#### Early era (pre-2010)

Studies from this period predominantly relied on plain radiography, myelography, and CT-myelography. Loembe et al. [26] employed the full complement of pre-MRI-era imaging in Gabon—AP, lateral, and oblique X-rays, dynamic views, plain CT, myelography, and CT-myelography. Andrews et al. [23] were compelled to use cervical myelograms and post-myelography CT because MRI was entirely unavailable in Ghana at the time of the study. Ogunniyi et al. [21] and Adeloye [29] provide insufficient imaging data in accessible sources to characterise the modalities used.

#### Transition era (2005–2015)

This period reflects the incremental adoption of CT and, subsequently, MRI, though access remained severely constrained in many settings. Kassegne et al. [27] documented CT-myelography (myeloscanner) in 90% of their Togolese cases, with only 5% accessing MRI, in a setting where no dedicated neuroimaging or neurosurgical service existed. Olarinoye-Akorede et al. [20] used a 0.2 Tesla MRI in Nigeria—a compromise between access and diagnostic resolution, noting that low-field strength may have underestimated cord pathology. Fidele & Amanuel [28] reported spinal MRI use in 74.3% of their total Ethiopian cohort, though this was not separately reported for the 16-patient cervical subgroup.

#### Recent era (2020–2025)

In the most contemporary cohorts, MRI became the predominant diagnostic modality. Ojo & Ikwuegbuenyi [14] and Elbhrawy et al. [30] both used MRI or CT-myelography as the primary diagnostic instrument. Figuim et al. [25] reported MRI in 76.92% of their Cameroonian series, with 23.07% receiving CT only—the latter predominantly in the earlier years of their 10-year study window. Mohammed et al. [31] employed both cervical MRI and high-resolution CT to characterise CPPD calcifications in their Egyptian cases. Elbhrawy et al. [30] used MRI as the standard preoperative planning tool in their Egyptian prospective cohort, including for compression level identification and surgical approach selection.

#### Key limitation: MRI scarcity and diagnostic delay

MRI scarcity was the single most consistently identified diagnostic barrier across all settings and eras. Multiple studies explicitly linked limited MRI access to delayed diagnosis, inadequate lesion characterisation, and worse neurological status at presentation. This was most starkly illustrated by the Ghanaian series [23], where the complete absence of in-country MRI necessitated myelography, and by Kassegne et al. [27] in Togo, where 5% MRI access reflected near-total dependence on CT-myelography. Even in more contemporary Cameroonian data [25], MRI remained unavailable for approximately one quarter of patients, and the low-field MRI study from Nigeria [20] underscored that physical access to an MRI scanner does not guarantee diagnostic-quality imaging. Across this review, limited MRI availability was consistently associated with delayed diagnostic evaluation and advanced neurological presentation. However, delayed presentation is likely multifactorial, additionally reflecting referral inefficiencies, financial barriers, healthcare access limitations, and delayed healthcare-seeking behaviour.

### Surgical management strategies

All surgical series included in this review performed decompressive surgery as the primary intervention. The surgical approach was determined by the level and laterality of compression, patient age, number of levels involved, surgeon experience, and available resources.

#### Anterior approaches

Anterior cervical discectomy and fusion (ACDF) was the predominant approach for anterior compressive pathology and in younger patients. Andrews et al. [23] performed non-instrumented ACDF in all 40 Ghanaian patients—a resource-adapted technique without plate fixation, with 90% of fusions at C4-5 and C5-6. Ojo & Ikwuegbuenyi [14] used ACDF in 51.2% of their Nigerian cohort. Elbhrawy et al. [30] applied ACDF in 52% of their Egyptian series, all under intraoperative neurophysiological monitoring (IONM). Loembe et al. [26] performed exclusively anterior procedures across all 18 Gabonese cases—including corporectomy, discectomy, and somatotomy with osteophytectomy—achieving solid fusion and stability in all patients at 3–5 months postoperatively.

#### Posterior approaches

Posterior decompressive laminectomy—with or without fusion or instrumented stabilisation—was the most commonly employed approach when pooled across all studies. Nyada et al. [24] performed a laminectomy in 79.5% of their Cameroonian cohort, identifying it as the most feasible option for multilevel disease. Figuim et al. [25] employed a posterior approach in 86.54% of their multicentre Cameroonian series. Fidele & Amanuel [28] performed a decompressive laminectomy in 62.5% (10/16) of their Ethiopian cervical subgroup. Mohammed et al. [31] performed posterior laminectomy with excision of calcified ligamentum flavum in both CPPD cases, with one patient additionally receiving lateral mass screw-rod arthrodesis.

#### Combined and resource-adapted strategies

**Fig. 5:**
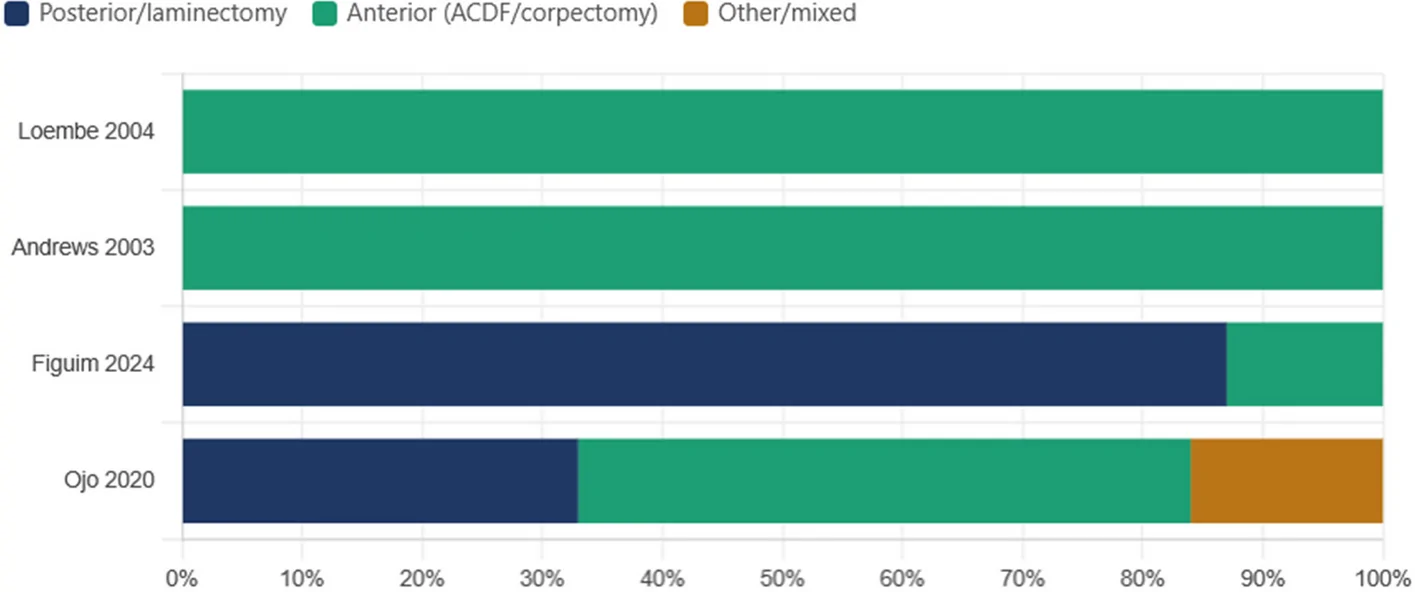
Surgical approach by study (%). Distribution of surgical approaches used across included studies (*n* = 15 studies; total patients = 315). Values represent proportions of procedures reported within each study. No statistical aggregation or confidence intervals were applied due to heterogeneity in study design and reporting methods

Combined anterior–posterior approaches were rarely described. Ojo & Ikwuegbuenyi [14] additionally performed decompression with lateral mass fixation (11.6%), corpectomy (2.3%), and occipitocervical fixation (2.3%), reflecting heterogeneous pathology at a Nigerian tertiary referral centre in which all surgical decisions were constrained by patient poverty, absence of operating microscopes, lack of C-arm fluoroscopy, and out-of-pocket implant costs. In Togo, Kassegne et al. [27] faced the most extreme resource limitation: the absence of any in-country neurosurgical service necessitated medical evacuation abroad for one patient, with a laminectomy performed within the existing Togolese hospital infrastructure for the remainder. IONM was reported in only one study—Elbhrawy et al. [30]—highlighting its near-complete absence across sub-Saharan African settings. Figure 5 summarises the surgical approach by study.

### Postoperative outcomes

#### Neurological improvement rates

Across all surgical series with extractable outcome data, postoperative neurological improvement was the dominant finding, with rates ranging from 50% to 97.6%:

Ojo & Ikwuegbuenyi (2020) [Nigeria]: 97.6% (41/43) demonstrated significant Nurick improvement postoperatively (*P* < 0.001). Mean Nurick grade declined from 3.2 preoperatively to 2.12 at 6 weeks and 1.14 at 6 months. Early presenters (< 3 months) achieved a mean Nurick of 0.5 at 6 months, versus 1.2 for late presenters (> 3 months). Loembe et al. (2004) [Gabon]: 10/18 (55.6%) achieved complete neurological recovery (Nurick 0-I); 7/18 (38.9%) achieved partial recovery (Nurick II); 1/18 remained unchanged (Nurick IV) at a mean follow-up of 6.9 years. Andrews et al. (2003) [Ghana]: 86% achieved excellent or good outcomes by Odom's criteria; mean Nurick grade improved from 2.3 to 1.3 postoperatively. Figuim et al. (2024) [Cameroon]: neurological improvement documented in 82% at 1 year; no progression in 8%; neurological worsening in 6%. Fidele & Amanuel (2015/2016) [Ethiopia]: surgical improvement in 50% (5/10) of operated cervical myelopathy patients—the lowest reported rate across surgical series, likely reflecting the more advanced neurological status of this Ethiopian cohort within a mixed aetiological setting.

Kassegne et al. (2013) [Togo]: 56% (5/9) showed clinical improvement during hospitalisation—the only available outcome measure given the absence of structured follow-up. Mohammed et al. (2025) [Egypt]: both CPPD cases improved to Nurick grade 1 postoperatively, from preoperative grades of 3 and 2, respectively. Guesmi et al. (2005) [Tunisia]: complete resolution of all symptoms following C3-C4 laminectomy and resection of calcified ligamentum flavum. Younes et al. (2008) [Tunisia]: significant reduction in pain and dysaesthesia postoperatively following multilevel laminectomy for fluorosis-associated OPLL, though motor deficits persisted.

#### Functional outcome scores

The Nurick grading system was the most frequently used functional outcome measure [14, 23, 25, 26, 31]. A Nurick improvement of 1–2 grades was the typical postoperative gain across these cohorts, with the most pronounced gains seen in early presenters [14].

The modified Japanese Orthopaedic Association (mJOA) score was used exclusively by Elbhrawy et al. [30]. mJOA did not significantly improve at 1 month (*P* = 0.051), but reached statistical significance at both 3 months and 12 months (*P* < 0.001 in both). Domain-specific recovery was greatest for upper limb motor function (65%), followed by lower limb motor function (52%), upper limb sensory function (48%), and sphincter function, which showed the least improvement. Preoperative severity was identified as the principal predictor of postoperative outcome.

Odom's criteria (excellent/good/fair/poor) was applied by Andrews et al. [23] as the sole outcome instrument—an older, non-validated categorical scale that limits comparability with contemporary cohorts.

### Predictors of better outcome

Several consistent prognostic factors for superior postoperative neurological recovery emerged across studies. Earlier presentation and shorter symptom duration were the most consistently identified predictors: Ojo & Ikwuegbuenyi [14] demonstrated that early presenters (< 3 months) achieved nearly twice the Nurick improvement of late presenters at 6 months, and Elbhrawy et al. [30] confirmed preoperative severity as the primary outcome determinant. MRI-based surgical planning was associated with more precise decompression and improved outcomes in both the Cameroonian [25] and Egyptian [30] series. Single or limited-level disease was implicitly associated with better outcomes across anterior approach series [23, 26]. Long-term durability of recovery was demonstrated by Loembe et al. [26], who showed sustained neurological improvement at a mean of 6.9 years, supporting durable benefit from timely anterior decompression and fusion.

### Follow-up duration

Follow-up varied considerably and was frequently limited or unreported. The majority of contemporary studies provide only short-term follow-up of ≤ 6 months: Ojo & Ikwuegbuenyi [14] reported at 6 weeks and 6 months; Elbhrawy et al. [30] at 1, 3, and 12 months. Figuim et al. [25] reported 1-year Nurick data for their full cohort, with a mean follow-up of 4 years (range 1–10 years) and 21% followed for ≥ 2 years, representing medium-term follow-up in a minority. Long-term follow-up (> 5 years) was documented in only one study: Loembe et al. [26], with a mean postoperative follow-up of 6.9 years (range 4–15 years). Multiple studies [21, 24, 25, 27, 29, 31–33], reported no structured postoperative follow-up, precluding any neurological trajectory analysis beyond the immediate perioperative period.

### Risk of bias synthesis

Using the JBI Critical Appraisal Checklist for case series and case reports, no included study achieved a low overall risk of bias judgement. Common methodological limitations across the body of evidence included:

Retrospective design dominance (> 70% of studies), precluding standardised prospective data collection and independent outcome ascertainment [20–27]. Lack of standardised outcome measures in older and lower-resourced studies, where Odom's criteria [23], non-validated clinical improvement narratives [27, 28], or no extractable outcome data whatsoever [21, 22, 29]were used in place of validated instruments.

Imaging heterogeneity across eras, with diagnostic sensitivity ranging from plain radiography [23] to low-field 0.2 T MRI [20] to modern 1.5 T MRI [30, 31], rendering cross-study severity comparisons unreliable. Severe financial selection bias in resource-limited settings, most explicitly documented by Kassegne et al. [27], in whom 73% of eligible patients were excluded from analysis because they could not afford diagnostic imaging, fundamentally biases the enrolled sample.

Incomplete follow-up reporting in more than half of the included studies [21, 22, 24, 27, 29, 31–33], preventing assessment of medium- or long-term neurological trajectories.

The two prospective cohort studies [14, 30] represented the highest-quality evidence within this review at moderate risk of bias. The multicentre Cameroonian series [25] was judged moderate-to-high risk. The three case reports [31–33] were assessed as very high risk given their inherently non-comparative, single- or two-patient design. The high degree of methodological heterogeneity, variability in aetiology, outcome instruments, and clinical settings across studies precluded formal meta-analysis; findings are therefore presented as a narrative synthesis throughout.

### Certainty of evidence

**Fig. 6:**
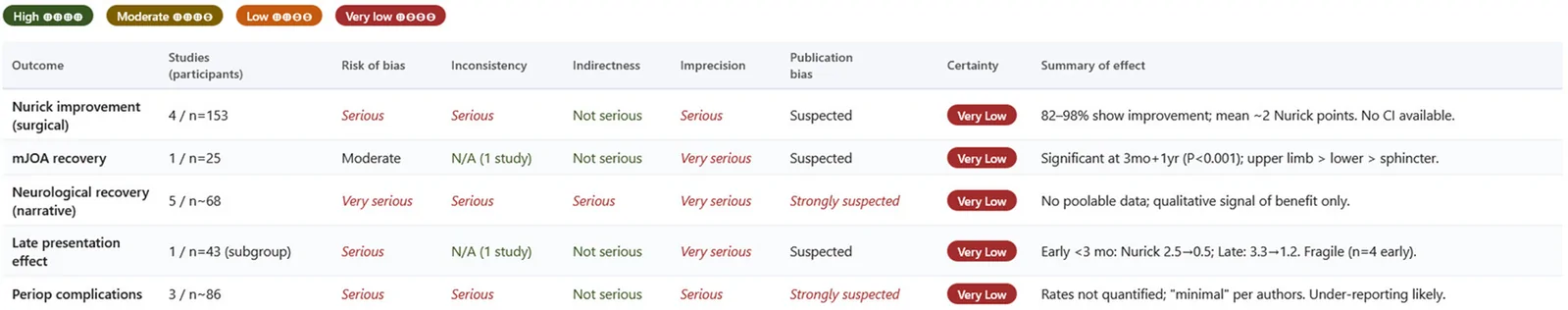
Summary of the certainty of evidence was evaluation using the GRADE framework. Summary of certainty of evidence using the GRADE framework across key outcomes. Certainty ratings reflect risk of bias, inconsistency, indirectness, and imprecision across included studies. No quantitative pooling or statistical error estimation is applicable

Using the GRADE approach, certainty of evidence was rated as very low for all outcomes because of methodological limitations, small sample sizes, inconsistent findings, and suspected publication bias. Figure 6 presents a visual summary of GRADE certainty assessments, while Table 2 provides a structured Summary of Findings with effect estimates and explicit downgrading decisions..

**Table 2:** Summary of findings and GRADE certainty assessment for cervical myelopathy in Africa

Outcome	Studies (n)	Participants (n)	Effect Estimate	Certainty of Evidence (GRADE)	Reasons for Downgrading
Postoperative neurological improvement (Nurick or equivalent)	4	153	82%–97.6% improved; mean ~ 2 Nurick grade gain	Very low	Serious risk of bias, inconsistency, imprecision, publication bias
Functional improvement (mJOA score)	1	25	Significant improvement at 3 and 12 months (p < 0.001)	Very low	Very serious imprecision, single-study evidence, publication bias
Overall neurological recovery (narrative synthesis)	5	~ 68	Reported improvement in majority of patients (50%–100%)	Very low	Very serious risk of bias, inconsistency, indirectness, imprecision
Early vs late presentation outcome difference	1	43	Early presenters: greater Nurick improvement (≈2.5 vs 1.2 grades)	Very low	Serious risk of bias, very serious imprecision, single study
Perioperative complications	3	~ 86	Complications variably reported; likely under-reported	Very low	Serious risk of bias, inconsistency, imprecision, publication bias

For surgical functional improvement measured by the Nurick scale, 4 studies (*n* = 153) reported postoperative improvement in 82–98% of patients, with a mean improvement of approximately 2 Nurick grades [14, 23, 25, 26]. Certainty was downgraded for serious risk of bias, inconsistency, and imprecision, with additional concern for publication bias, resulting in very low-certainty evidence.

Recovery assessed using the mJOA score was reported in a single study (*n* = 25), which demonstrated significant improvement at ≥ 3 months and 1 year follow-up (*P* < 0.001). Certainty was downgraded for very serious imprecision and suspected publication bias, yielding very low-certainty evidence [30].

Neurological recovery outcomes from 5 narrative studies (n≈68) suggested postoperative neurological benefit; however, no pooled quantitative estimate was possible. Certainty was downgraded for very serious risk of bias and imprecision, serious inconsistency and indirectness, and strongly suspected publication bias, resulting in very low-certainty evidence [26–28, 31, 33]

For the late-presentation subgroup, 1 study (n≈43) reported smaller improvements among patients presenting within < 3 months compared with later presentation (Nurick improvement: 2.5 → 0.5 vs 3.3 → 1.2). Because subgroup numbers were small and estimates unstable, certainty was downgraded for serious risk of bias and very serious imprecision, resulting in very low-certainty evidence [14].

Perioperative complications were described in 3 studies (n≈86), but complication frequencies were incompletely quantified and likely under-reported [14, 24, 25]. Certainty was downgraded for serious risk of bias, inconsistency, imprecision, and strongly suspected publication bias, resulting in very low-certainty evidence. Figure 6 presents a summary of GRADE outcome-level certainty assessments.

## Discussion

### Principal findings

This systematic review of 15 studies encompassing approximately 350–400 patients with cervical myelopathy across the African continent demonstrates that degenerative cervical spondylotic myelopathy (CSM) constitutes the predominant aetiology in surgical cohorts. Patients consistently present with advanced disease, characterised by Nurick grades predominantly ≥ 3 or equivalent severity on modified Japanese Orthopaedic Association (mJOA) scoring, following prolonged symptom duration ranging from 12 to 48 months. Core neurological features include motor deficits (particularly spastic tetraparesis) and gait disturbance in the large majority of cases, with sensory symptoms variably reported and sphincter dysfunction emerging as a late manifestation. Surgical decompression, delivered through resource-adapted anterior or posterior approaches, yields neurological improvement in 50–97% of patients with meaningful gains on Nurick and mJOA scales, particularly in those with shorter symptom duration. Diagnostic imaging has evolved from reliance on plain radiography and myelography to increasing utilisation of MRI, although access constraints remain evident even in contemporary series.

### Interpretation of findings

The near-universal pattern of advanced myelopathy at presentation across West, Central, East, and North African cohorts reflects systemic healthcare barriers, including limited MRI availability, financial constraints, and inadequate referral pathways, rather than fundamental differences in disease biology. Loembe et al. [26], Nyada et al. [24], Ojo & Ikwuegbuenyi [14], and Figuim et al. [25] consistently documented prolonged mean symptom durations and high preoperative Nurick grades, with early presenters achieving substantially better functional recovery. This delayed health-seeking behaviour explains the high frequency of multilevel degenerative compression and the prominence of gait impairment and motor deficits as presenting features.

Degenerative CSM predominated across all major surgical series [14, 23–26, 30], mirroring the global epidemiology of the condition, while rarer aetiologies such as calcium pyrophosphate deposition disease (CPPD)-related ligamentum flavum calcification [31, 32] and skeletal fluorosis with ossification of the posterior longitudinal ligament [33] retain regional importance. Posterior laminectomy served as the most frequently employed strategy for multilevel disease [24, 25], whereas anterior cervical discectomy and fusion (ACDF) was favoured for focal anterior pathology [14, 23, 26, 30]. These tailored, resource-adapted techniques produced consistent neurological improvement despite minimal use of instrumentation or intraoperative neuromonitoring, suggesting potential neurological benefit following timely decompression, even under constrained conditions. Preoperative severity and symptom duration emerged as the principal modifiers of outcome, aligning with the known natural history of progressive cord compression.

### Comparison with existing literature

The aetiological predominance of degenerative CSM and the benefit of surgical decompression observed in this African synthesis align with international evidence. Recent systematic reviews and meta-analyses support that surgery is associated with better functional recovery compared with conservative treatment in CSM, with clinically meaningful improvement in mJOA scores [34]. Global series typically report earlier presentation and lower baseline severity than the African cohorts, where mean symptom duration often exceeds 12–45 months, and Nurick grades ≥ 3 are common [34, 35]. In high-resource settings, mean preoperative mJOA are frequently higher, and patients achieve greater absolute gains, with widespread use of instrumentation and neuromonitoring [35].

Despite the disparity in resources, postoperative improvement rates in the African series (50–97.6%) are comparable to those reported in recent reviews of the anterior and posterior approaches [35]. The prognostic significance of a shorter duration of symptoms and a lower preoperative severity, as emphasised by Ojo & Ikwuegbuenyi (2020 and Elbhrawy et al., is consistent with broader literature identifying these as key determinants of recovery. Limited long-term follow-up in most African studies contrasts with series demonstrating sustained benefit beyond 5 years in optimised settings [36, 37]. In sub-Saharan settings, the late presentation pattern is mirrored in reports on non-traumatic myelopathies, where diagnostic delays similarly compromise outcomes [10]. These African results further confirm the utility of core decompressive surgery but highlight the outcome disparity due to access to care rather than surgical technique itself.

### Strengths and limitations

Strengths of this review include its status as the first continental synthesis of cervical myelopathy, capturing temporal trends in imaging and surgical practice across four sub-regions and incorporating both predominant degenerative cases and rarer aetiologies. Narrative synthesis effectively highlights consistent clinical and outcome patterns despite study heterogeneity.

Limitations are inherent to the primary evidence. More than 70% of included studies employed retrospective or cross-sectional designs, conferring high risk of bias [23–26]. Outcome measures and follow-up duration varied considerably, with most contemporary series limited to ≤ 12 months and only Loembe et al. [26] providing a mean follow-up beyond 5 years. Financial selection bias, incomplete reporting, and imaging heterogeneity (from myelography to low-field MRI) further reduce comparability. No study achieved a low overall risk of bias, and methodological diversity precluded meta-analysis. “Potential overlap between institutional cohorts from Cameroon and Nigeria could not be fully excluded because several studies originated from similar tertiary referral settings with partially overlapping study periods. At the review level, the absence of eligible Southern African data and reliance on published literature represent important gaps. Publication bias likely substantially influences the available evidence base. Most included studies originated from tertiary neurosurgical centres and predominantly reported surgically treated patients with favourable postoperative outcomes. Failed decompressions, conservatively managed patients, untreated advanced disease, perioperative mortality, and patients unable to access surgery may therefore be underrepresented in published literature, potentially inflating perceived treatment effectiveness.

### Implications for practice and policy

These findings support the feasibility and effectiveness of surgical decompression for cervical myelopathy across diverse African settings. Clinical practice should prioritise early referral once symptoms such as gait disturbance or tetraparesis appear, alongside adoption of resource-adapted protocols (e.g., non-instrumented ACDF or laminectomy for multilevel disease). Health systems should focus on expanding MRI access, optimising low-field scanners, strengthening referral pathways, and mitigating financial barriers to reduce preoperative disability and maximise recovery. Standardised use of Nurick and mJOA scores would facilitate future benchmarking and quality improvement.

### Future research directions

Future research should prioritise well-designed prospective multicentre registries that employ standardised diagnostic criteria, consistent timing metrics (symptom onset to surgery), validated outcome measures (Nurick and mJOA), and extended long-term follow-up. There is a particular need to generate data from Southern Africa, which was entirely unrepresented in the current evidence base.

Additional priorities include evaluation of cost-effective innovations such as optimised low-field MRI protocols, affordable spinal implants, and task-shifting models. Studies investigating modifiable risk factors for both degenerative CSM and non-degenerative causes of cervical myelopathy (e.g., fluorosis, CPPD) in African populations are also warranted. Such efforts will be essential to inform context-specific guidelines and reduce the burden of preventable disability from cervical myelopathy across the continent.

## Conclusion

Available evidence suggests that surgical decompression is feasible in African settings and may provide meaningful neurological improvement in selected patients. However, interpretation is limited by retrospective study designs, heterogeneous outcome measures, incomplete follow-up, and likely publication bias. Outcomes are strongly influenced by preoperative disease severity and symptom duration. Strengthening early referral pathways and improving access to diagnostic imaging may improve functional outcomes across the continent.

## Supplementary Information

Supplementary Material 1.

## Data Availability

All data generated and analysed during this systematic review are included in this published article and its supplementary materials. No new datasets were generated. All extracted data are available from the original published studies referenced in this review.

## Abbreviations

ACDF: Anterior Cervical Discectomy and Fusion
AJOL: African Journals Online
CPPD: Calcium Pyrophosphate Deposition Disease
CSM: Cervical Spondylotic Myelopathy
CT: Computed Tomography
DCM: Degenerative Cervical Myelopathy
GRADE: Grading of Recommendations Assessment, Development and Evaluation
IONM: Intraoperative Neurophysiological Monitoring
JBI: Joanna Briggs Institute
LMICs: Low- and Middle-Income Countries
mJOA: Modified Japanese Orthopaedic Association
MRI: Magnetic Resonance Imaging
OPLL: Ossification of the Posterior Longitudinal Ligament
PRESS: Peer Review of Electronic Search Strategies
PRISMA: Preferred Reporting Items for Systematic Reviews and Meta-Analyses
