# Supplementary material for: Cervical compressive myelopathy in Africa: a systematic review of clinical presentation, diagnostic pathways, surgical management, and outcomes
Source: BMC Neurol. 2026 Jun 29;26:582. doi: 10.1186/s12883-026-05106-x (PMC13576288; doi:10.1186/s12883-026-05106-x)
Supplement: Supplementary file 1 [file 12883_2026_5106_MOESM1_ESM.docx]

# **Supplementary File 1: Full Electronic Search Strategies**

### **Search Development and Information Sources**

The search strategy was developed to maximise sensitivity for studies reporting cervical compressive myelopathy in African settings. Search concepts combined controlled vocabulary terms (Medical Subject Headings [MeSH], where applicable) with free-text keywords relating to:

1. Cervical myelopathy and cervical spinal cord compression
2. Degenerative and non-degenerative compressive cervical pathology
3. Surgical management and neurological outcomes
4. African geographic settings

The search strategy was iteratively refined and peer-reviewed in accordance with the PRESS (Peer Review of Electronic Search Strategies) framework. Search reporting additionally followed PRISMA-S recommendations where applicable.

Electronic database searches were conducted in:

- PubMed/MEDLINE
- African Journals Online (AJOL)
- Google Scholar

Additional hand-searching of reference lists from eligible studies and relevant review articles was also performed.

The final search update was conducted on 25th December, 2025. Searches were limited to studies involving human subjects. Publications in English or French were eligible for inclusion.

# **1. PubMed/MEDLINE Search Strategy**

**Database:** PubMed/MEDLINE (National Library of Medicine)
**Date searched:** 25th December, 2025

### **Search Strategy**

(

"Cervical Vertebrae"[Mesh]

OR cervical[Title/Abstract]

)

AND

(

"Myelopathy"[Title/Abstract]

OR "cervical myelopathy"[Title/Abstract]

OR "degenerative cervical myelopathy"[Title/Abstract]

OR "cervical spondylotic myelopathy"[Title/Abstract]

OR "compressive cervical myelopathy"[Title/Abstract]

OR "spinal cord compression"[Title/Abstract]

OR "cervical cord compression"[Title/Abstract]

OR "ossification of the posterior longitudinal ligament"[Title/Abstract]

OR OPLL[Title/Abstract]

OR "ligamentum flavum calcification"[Title/Abstract]

)

AND

(

"Africa"[Mesh]

OR Africa[Title/Abstract]

OR "sub-Saharan Africa"[Title/Abstract]

OR Nigeria[Title/Abstract]

OR Ghana[Title/Abstract]

OR Cameroon[Title/Abstract]

OR Gabon[Title/Abstract]

OR Togo[Title/Abstract]

OR Ethiopia[Title/Abstract]

OR Egypt[Title/Abstract]

OR Tunisia[Title/Abstract]

OR Malawi[Title/Abstract]

)

AND

(

"Spinal Fusion"[Mesh]

OR "Decompression, Surgical"[Mesh]

OR surgery[Title/Abstract]

OR decompression[Title/Abstract]

OR laminectomy[Title/Abstract]

OR ACDF[Title/Abstract]

OR "anterior cervical discectomy and fusion"[Title/Abstract]

OR neurosurgery[Title/Abstract]

OR "spinal surgery"[Title/Abstract]

OR outcome[Title/Abstract]

OR recovery[Title/Abstract]

OR Nurick[Title/Abstract]

OR mJOA[Title/Abstract]

)

### **Filters Applied**

- Humans
- English OR French language

### **Records Retrieved**

n = 88

# **2. African Journals Online (AJOL) Search Strategy**

**Database:** African Journals Online (AJOL)
**Date searched:** 25th December, 2025

Because AJOL has limited advanced Boolean search functionality compared with PubMed, multiple simplified keyword combinations were used.

### **Primary Search Terms**

"cervical myelopathy"

"degenerative cervical myelopathy"

"cervical spondylotic myelopathy"

"compressive cervical myelopathy"

"spinal cord compression"

"cervical cord compression"

### **Geographic Search Terms**

Africa

"sub-Saharan Africa"

Nigeria

Cameroon

Ghana

Togo

Ethiopia

Egypt

Tunisia

Malawi

### **Additional Surgical and Outcome Terms**

"spinal surgery"

"decompression surgery"

laminectomy

ACDF

fusion

outcome

recovery

Nurick

mJOA

Reference lists of eligible AJOL-indexed studies were manually screened for additional publications.

### **Records Retrieved**

No additional eligible studies were identified exclusively through AJOL searches beyond those captured in PubMed and Google Scholar.

# **3. Google Scholar Search Strategy**

**Database:** Google Scholar
**Date searched:** 25th December, 2025

Google Scholar searches were conducted using broad free-text combinations to maximise sensitivity for regional, non-indexed, and institutionally archived African neurosurgical literature.

### **Primary Search Strings**

"cervical myelopathy" Africa

"degenerative cervical myelopathy" Africa

"cervical spondylotic myelopathy" Africa

"compressive cervical myelopathy" Africa

"spinal cord compression" cervical Africa

"cervical decompression surgery" Africa

### **Country-Specific Searches**

Nigeria cervical myelopathy

Cameroon cervical myelopathy

Ghana cervical myelopathy

Ethiopia cervical myelopathy

Egypt cervical myelopathy

Tunisia cervical myelopathy

Malawi cervical myelopathy

### **Screening Approach**

Because Google Scholar returns highly dynamic search outputs, searches were conducted using relevance ranking. Screening was restricted to the first 200 relevance-ranked records for each major search string, consistent with methodological guidance for systematic reviews using Google Scholar.

Titles and abstracts were screened for relevance, and duplicate citations and non-original publications were excluded prior to eligibility assessment.

### **Records Retrieved**

n = 4660

### **Methodological Limitation**

Google Scholar searches may have limited reproducibility because of continuously changing indexing algorithms and relevance-ranking processes.

**Supplementary Search Methods**

## **Hand-searching**

Reference lists of all eligible studies and relevant review articles were manually screened to identify potentially eligible additional studies.

## **Grey Literature and Registry Searches**

Targeted searches of African neurosurgical and spinal surgery sources were conducted to identify additional eligible studies and institutional reports. No additional eligible unpublished datasets meeting inclusion criteria were identified.

## **Search Management**

All retrieved citations were exported into Rayyan for duplicate removal and screening prior to eligibility assessment.

# **PRISMA Flow Summary**

- Records identified from database searches: n = 4748
  - Google Scholar: n = 4660
  - PubMed/MEDLINE: n = 88
- Duplicate records removed before screening: n = 1088
- Records screened: n = 3660
- Records excluded during title/abstract screening: n = 3594
- Reports sought for retrieval: n = 66
- Reports not retrieved: n = 6
- Full-text reports assessed for eligibility: n = 60
- Full-text reports excluded: n = 45
  - Non-original publications: n = 24
  - Non-cervical compressive myelopathy studies: n = 15
  - Studies without extractable outcome data: n = 6
- Studies included in qualitative synthesis: n = 15
- Reports of included studies: n = 15
